# Prevalence and Treatment Outcomes of Childhood Acute Lymphoblastic Leukemia in Kosovo

**DOI:** 10.3390/cancers16111988

**Published:** 2024-05-23

**Authors:** Flaka Pasha, Dunja Urbančič, Rufadie Maxhuni, Shaip Krasniqi, Violeta Grajçevci Uka, Irena Mlinarič-Raščan

**Affiliations:** 1 Faculty of Pharmacy, University of Ljubljana, Askerceva 7, 1000 Ljubljana, Slovenia; flaka.pasha@uni-pr.edu (F.P.); dunja.urbancic@ffa.uni-lj.si (D.U.); 2 Department of Pharmacology with Toxicology, and Clinical Pharmacology, Faculty of Medicine, University of Prishtina “Hasan Prishtina”, 10000 Prishtine, Kosovo; 3 Department of Hematology-Oncology, Pediatric Clinic, University Clinical Center of Kosovo, 10000 Prishtine, Kosovo

**Keywords:** acute lymphoblastic leukemia, treatment, overall survival, event-free survival, pediatric

## Abstract

**Simple Summary:**

This is the first report portraying the situation of childhood acute lymphoblastic leukemia (ALL) in Kosovo over the past 15 years. We examine treatment protocols, remission and relapse rates, and 2-year and 5-year event-free survival (EFS) and overall survival (OS) rates. In our study cohort, 55% of patients achieved 2-year EFS, 40% achieved 5-year EFS, 61% achieved 2-year OS, and 46% achieved 5-year OS, which are lower rates compared to most other studies. These findings emphasize the importance of genotyping for common polymorphisms and therapeutic drug monitoring, which have not been utilized to date, as foundational methods among healthcare providers treating childhood ALL in Kosovo.

**Abstract:**

Advances in research, including novel biomarker identification and patient stratification, have significantly improved the therapy for childhood acute lymphoblastic leukemia (ALL), though access to improved healthcare services varies across geographical regions. In an effort to evaluate the advances in therapeutic approaches, we performed a retrospective analysis of childhood ALL in Kosovo. Our retrospective analysis included 225 cases diagnosed between 2008 and 2023, representing 52% of 429 diagnosed childhood cancers. The average annual incidence was 14, with a median age diagnosis of seven years, and a male predominance (59.54%). Patients were categorized into risk groups, with the majority (43%) in the standard-risk category. We identified five different treatment protocols for this study period. Over 61% of patients achieved remission after the first chemotherapy cycle and we observed a 20% mortality rate. Survival analysis showed that 55% and 40% of patients achieved 2-year and 5-year event-free survival (EFS), respectively, with significant differences across risk groups. Treatment advancements significantly correlated with improved survival rates, achieving a 5-year overall survival (OS) of 88% in the currently used standardized AIEOP-BFM-2009 protocol. Our study emphasizes the need for continued research and customized care strategies to enhance clinical outcomes.

## 1. Introduction

Acute lymphoblastic leukemia (ALL) is the most common malignant disease in childhood, accounting for 25.3% of all newly diagnosed childhood cancers [1]. In the United States, the annual incidence is 3.7 to 4.9 cases per 100,000 children [2]. The risk of developing ALL is slightly higher in males than in females [3], with the incidence being highest between the ages of 2 and 5 years [4]. The etiology of the disease remains unknown, although environmental, immunologic, socioeconomic, and epidemiologic factors have been associated with childhood ALL. Contributing factors to leukemogenesis thus include previous exposure to ionizing radiation [5], chemotherapy, or radiation therapy [6]; Down syndrome [7]; ataxia-telangiectasia; Fanconi anemia; Li–Fraumeni syndrome; neurofibromatosis 1; or other genetic disorders [8].

The diagnosis and characterization of childhood ALL are based on a complete blood count and biochemical blood tests, chest X-ray, bone marrow aspiration biopsy, cytogenetic analysis, and immunophenotyping [9]. Childhood ALL progresses rapidly if not treated effectively and can lead to death. Due to the increased number of immature blasts that replace normal blood cells in the blood marrow, the disease manifests itself in bruising, anemia, and frequent infections as early clinical signs. Fever, painless swollen lymph nodes, loss of appetite, fatigue, and shortness of breath may also occur as less specific features [10].

Advances in biomarkers have enabled precision medicine approaches in diagnostics and the prediction of patient response to therapies. Therapy based on clinical, biologic, and genetic features has led to observed improvement in survival rates, which currently exceed 90% in high-income countries. In contrast, the estimated survival in low- and middle-income countries ranges from 22 to 79%, depending on the region and local resources [11,12,13].

The basic framework of treatment for childhood ALL includes induction of remission with chemotherapy starting at the time of diagnosis, followed by consolidation, delayed intensification, and maintenance therapy [14]. Treatment protocols for ALL have improved dramatically from aminopterin, which achieved only a temporary remission in 1948 [15], to the chemotherapy regimens used today, which have been refined through several consolidative clinical experiences of the AIEOP and BFM study groups. On account of the improved regimens, current treatment of ALL now achieves a 5-year survival rate of 92% [16].

Despite the immense efforts made over the last 60 years to improve and cure childhood ALL, little is known about the epidemiology and treatment of childhood ALL in many countries [17]. Thus, epidemiologic and clinical research studies are needed in countries and regions less represented in current reports. In this retrospective study, we provide the first report on the prevalence and treatment outcomes of childhood ALL in the Republic of Kosovo. The data were collected from records of childhood ALL patients treated at the Department of Pediatric Hematology-Oncology, University Clinical Center of Kosovo, over the past 15 years and analyzed for prevalence and treatment outcomes.

## 2. Materials and Methods

### 2.1. Data Collection

In this retrospective cohort study, data were extracted from patients’ charts of childhood ALL patients treated at the Department of Pediatric Hematology-Oncology, University Clinical Center of Kosovo, which is the only referral center for patients with ALL in the country. Epidemiological data for overall childhood malignancies were obtained from the oncology database of the Department of Pediatric Hematology-Oncology and the cancer registry of National Institute of Public Health. This study was approved by the Ethics Committee of Faculty of Medicine, University of Prishtina “Hasan Prishtina” in Kosovo (approval No. 4095), and the University Clinical Center of Kosovo (approval No. 1429). We confirm that this research was conducted in accordance with the World Medical Association Declaration of Helsinki, with careful consideration of ethical principles for medical research involving human subjects.

Collected data spanned from March 2008 to September 2023 and resulted in a total of 225 ALL patients who were younger than 18 years old at the time of diagnosis. We recorded demographic data, comorbidities at the time of diagnosis, treatment protocol, remission after the first cycle of chemotherapy, relapse, most frequent sites of relapse, referral rates to regional and international specialty centers for hematopoietic stem cell transplantation, loss to follow-up, and mortality due to primary diagnoses or complications. From the data, we extracted 2-year and 5-year event-free survival (EFS) rates and overall survival (OS) rates stratified by age groups, risk groups, gender, remission after the first cycle of chemotherapy, and different treatment protocols. The information obtained from the records was taken from the relevant physicians at the time of diagnosis, and the information was provided by the child’s caregiver(s).

### 2.2. Risk Determination

Patients were categorized into very high-, high-, standard-, and low-risk groups based on the Pediatric Oncology Group (POG) and Children’s Cancer Group (CCG) risk classification for childhood ALL [18,19]. Risk factors included the following: age < 1 year old or age > 10 years old, white blood cell (WBC) count > 50 × 10^9^ cells/L at time of diagnosis, extra medullary disease, biologic and cytogenetic changes such as Philadelphia chromosome, T-ALL, positive cerebrospinal fluid (CSF) and testicular involvement, inability to tolerate standard chemotherapy, slow-rate response to initial therapy, minimal residual disease (MRD), and bone marrow aspiration. Children with hereditary comorbidities such as Down syndrome and cystic fibrosis were also categorized into risk groups based on guidelines from POG and CCG.

### 2.3. Treatment Protocols

**Figure 1:**
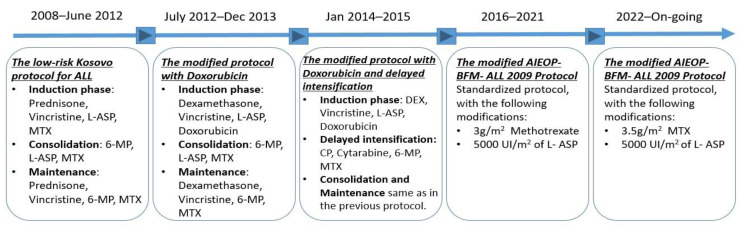
Timeline chart of childhood acute lymphoblastic leukemia (ALL) treatment protocols followed in Kosovo from 2008 to 2023.

During the investigated time period, five different treatment protocols were used to treat children with ALL (Figure 1). Detailed information on treatment protocols is presented in Supplementary Information, SI—Detailed childhood ALL treatment protocols followed in Kosovo from 2008 to 2023 [20].

### 2.4. Data Analysis

Data were analyzed using SPSS version 29. Data on summarized demographic characteristics, clinical profiles, treatment protocols, and risk groups are presented as means and standard deviations or as frequencies and percentages using descriptive statistics. The chi-squared test was used to analyze differences between groups. The log-rank test was used to estimate the OS and EFS rates. Statistical significance was evaluated at an alpha level (α) of 0.05.

## 3. Results

### 3.1. Characteristics of the Sample and Incidence of Childhood ALL in Kosovo

Our study identified 225 cases of childhood ALL among the 429 childhood malignancies diagnosed in Kosovo from 2008 to 2023, with ALL constituting 52% of total malignancies in the pediatric population of Kosovo (Table 1). The average annual incidence was 14, reaching a peak of 22 cases in 2017 and a low of 7 cases in 2019. All patients included in this study had type B ALL. The average age at diagnosis was 7 years, with notable peaks at ages 4 and 12. There were 98 patients (43%) younger than 5 years, 64 patients (28%) in the age group of 5–10-year-olds, and 63 patients (28%) older than 10 years (Table 1). There were more males (133, 59.54%) than females (91, 40.44%). No significant differences were observed between genders across different age groups (chi-squared test; *p* = 0.1537). In the cohort, three patients with Down syndrome were concurrently diagnosed with childhood ALL, comprising two males aged 13 and one female aged 5. Additionally, a 6-year-old male with cystic fibrosis was concomitantly diagnosed with childhood ALL (Table 1).

According to the POG and CCG risk classification, patients diagnosed with childhood ALL were stratified into very high-, high-, standard-, and low-risk groups [18,19]. The low-risk group criteria were as follows: age < 10 years old, WBC count < 50,000/µL, and exhibiting either the t(12;21), (TEL/AML1) translocation, or simultaneous trisomy of chromosomes 4, 10, and 17. The standard-risk group criteria were as follows: age < 10 years old, WBC count < 50,000/µL, presenting with triple trisomy or TEL-AML1, and having less than 5% blasts in bone aspirate on days 8, 15, and 29. Additionally, they exhibited CNS stage 2 with blasts in the cerebrospinal fluid (CSF) and <5 nucleated cells/µL, along with testicular disease. The high-risk group criteria were as follows: age > 10 years old, WBC count > 50,000/µL, displaying MLL translocation, bone aspirate showing 5–25% blasts on day 15, manifesting stage 3 CNS changes with ≥5 nucleated cells/µL and ALL blasts detected by cytospinning, and active testicular disease. The very-high-risk group criteria were as follows: evidence of t(9;22) or BCR/ABL fusion, MLL translocation with a SER, and manifested induction failure with >25% blasts after bone marrow aspirate on day 29, irrespective of cellularity [19].

The distribution in our cohort was as follows: 35 patients (16%) in the very high-risk group, 81 patients (36%) in the high-risk group, 98 patients (43%) in the standard-risk group, and 11 patients (5%) in the low-risk group (Table 1).

Next, we analyzed whether the severity of the disease correlates with other variables such as age, gender, and comorbidities (Table 2). The standard-risk group primarily included patients aged 0–5 years, accounting for 56 cases (25%). In contrast, the high- and very high-risk groups mostly comprised patients over 10 years old, with 41 patients (18%) and 16 patients (7%), respectively. The low-risk group, consisting of only 11 patients, was evenly distributed across the three age groups. Significant differences were observed among risk groups and age groups (Table 2; chi squared test; *p* < 0.001). A Bonferroni post hoc test revealed significant differences within age groups 0–5 and >10 for standard- and high-risk groups. However, no statistically significant differences were found between risk groups in relation to gender and comorbidities. 

### 3.2. Diverse Treatment Protocols Were Followed Consecutively for Fifteen Years

Over the 15-year period, various protocols for childhood ALL treatment were implemented in Kosovo (Figure 1). Even though childhood ALL patients were stratified in different risk groups based on age, white blood cell count, gender, extra medullary spread of the disease, blast cytogenetics, ploidy, and early response to therapy, all patients received uniform treatment at the Hematology-Oncology Department in Pediatric Clinic at the University Clinical Center of Kosovo during specific periods of time. The current protocol is the modified AIEOP-BFM 2009 with 3.5 g/m^2^ of methotrexate [21]. 

A number of patients were referred to treatments in other international centers. Among the 225 children diagnosed with ALL in Kosovo during the 15-year period, 138 patients (61%) were treated exclusively in Kosovo, 13 patients (6%) started treatment in Kosovo and then continued abroad, and 74 patients (33%) were referred to regional and international medical centers for reasons such as immediate need for hematopoietic stem cell transplantation, having been categorized into a very high-risk group, advanced stage of disease, or greater need for more specialized care. The main referral centers were Turkey, with 40 patients (54%), followed by Italy with 14 patients (18%), and Austria with 8 patients (11%) (Table 3).

Overall, there were 151 patients diagnosed and treated in Kosovo or in Kosovo and abroad combined. Treatment followed the five different protocols detailed in the Materials and Methods section. Forty-three patients were treated under the low-risk Kosovo protocol for childhood ALL, sixteen patients were treated under the modified protocol with doxorubicin, twelve patients were treated under the modified protocol with doxorubicin + delayed intensification, fifty-eight of the patients were treated with the modified AIEOP-BFM 2009 protocol with 3 g/m^2^ of methotrexate, and twenty-two patients were treated with the modified AIEOP-BFM 2009 protocol with 3.5 g/m^2^ of methotrexate (Table 3). Patients who started their treatment with a particular protocol followed the same protocol until the completion of their therapy, even if the institution introduced a new protocol for newly diagnosed patients. Additional information about further classification of patients into risk groups in relation to their treatment protocols and country of treatment is provided in the Supplementary Information (Supplementary Materials Table S1) [20].

### 3.3. Remission, Relapse, and Mortality Rates of Childhood ALL in Kosovo

To contribute to the understanding of the efficacy of therapeutic approaches, we next evaluated the remission rates, relapse rates, and mortality rates of childhood ALL in Kosovo. Remission was defined as less than 5% immature blasts remaining in bone marrow after the first cycle of chemotherapy [22]. We evaluated remission rates after the first cycle of chemotherapy, relapse rates and sites, secondary infiltrations, and infections.

Of the 225 patients diagnosed, 22 patients (10%) were lost to follow-up (Table 4). Among the 203 monitored cases, 23 patients (11%) relapsed primarily in the bone marrow (76%), followed by the testes, retina, and central nervous system (8% each). In addition, 19 patients (9%) required hematopoietic stem cell transplantation (Table 4). Over 61% of the monitored patients (n = 123) achieved full remission after the first cycle of chemotherapy. First and second remissions both had 18 patients (9%). 

The overall mortality rate was 20% (28 cases) in patients treated in Kosovo (n = 141) and 25% (15 cases) in those treated abroad (n = 60) (Table 4). Childhood ALL as the primary diagnosis was the cause of death in 13% of the cases treated in Kosovo (n = 18), while 7% of patients died due to the secondary complications such as sepsis, acute respiratory distress syndrome, thrombophlebitis, meningitis, intracranial bleeding, pseudomonas aeruginosa infection, liver failure, paralytic ileus, and parotitis (Table 4).

We further analyzed whether mortality rates correlate with variables like age, gender, treatment protocol, and risk groups. The correlation between mortality rates and treatment protocols was statistically significant (*p* < 0.001). The highest mortality rate was observed in the high-risk and very high-risk groups. In this cohort, there were no statistically significant differences in mortality rates across age groups, genders, or the place of treatment. Additional information about further classification of mortality rates in relation to their risk group and treatment protocols is provided in the Supplementary Information (Supplementary Materials Tables S2 and S3) [20].

### 3.4. Event-Free and Overall Survival

Lastly, in our study of childhood ALL in Kosovo, we evaluated therapeutic outcomes through 2-year and 5-year OS and EFS rates. OS was calculated from the diagnosis to the last follow-up day or death, and EFS was considered as the period from diagnosis to the first event, such as relapse, secondary infiltration, infection, complications, lost to follow-up, or death. Of all patients (*N* = 225), 55% (*n* = 123) achieved 2-year EFS, and 40% (*n* = 90) achieved 5-year EFS. For OS, 61% of patients (*n* = 138) achieved a 2-year OS, and 46% (*n* = 104) achieved a 5-year OS.

**Figure 2:**
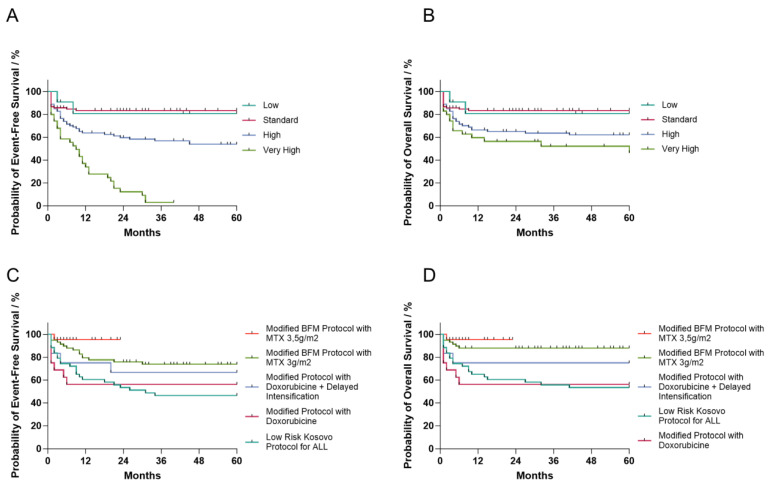
Five-year event-free and overall survival probabilities for patients with acute lymphoblastic leukemia (ALL) treated in Kosovo compared across different risk groups (**A**,**B**) and treatment protocols (**C**,**D**). Differences across risk groups and treatment protocols are statistically significant (log-rank test for trend, *p* < 0.001). Bars represent censored data.

Our study conducted 2-year and 5-year EFS and OS analyses using Kaplan–Meier curves to assess these rates across different risk groups, age groups, gender, and treatment protocols. Highly statistically significant differences were observed in 2-year and 5-year EFS and OS rates when comparing risk groups. Notably, the low- and standard-risk groups exhibited the highest survival rates, whereas those in the high-risk group had the lowest. Specifically, the low- and standard-risk groups had over an 80% probability of achieving 5-year EFS and OS. However, the high- and very high-risk groups had unexpectedly low 5-year EFS, with probabilities of 54% and less than 5%, respectively (Figure 2A). Their 5-year OS probabilities were around 62% and 46%, respectively, while the low- and standard-risk groups had 5-year OS probabilities of 81% and 83%, respectively (Figure 2B). No statistically significant differences were observed in survival rates when analyzed by age group or gender (Table 5). 

In the treatment protocols of patients treated only in Kosovo, there is a noticeable trend of increasing survival rates over time, correlating with the advancement of protocol modification implemented between 2008 and 2023. Our study showed statistically significant differences in 2-year and 5-year OS rates and in 5-year EFS rates. The probability of achieving 5-year EFS was 47% and 74% for the low-risk Kosovo protocol and the AIEOP-BFM-2009 protocol with methotrexate doses lowered to 3 g/m^2^, respectively (Figure 2C). Patients treated under the low-risk and modified protocols with doxorubicin had the lowest 5-year OS probability (53%), while patients treated under the standardized AIEOP-BFM-2009 protocol with methotrexate doses lowered to 3 g/m^2^ had a 5-year OS of 88% (Figure 2D). The differences in 2-year EFS were not significant (Table 5). The survival curves for all other parameters (2-year and 5-year EFS and OS for age, risk group and treatment protocol) can be found in SI Figures S3 and S4 [20].

## 4. Discussion

Childhood ALL contributed to over 52% of pediatric malignancies in Kosovo between 2008 and 2023, resembling the prevalence in other developing countries [23]. In our study, ALL prevalence was greater for males (60%) than females, similar to other studies [24,25,26]. The average age at diagnosis in our study group was 7 years old, which is higher than the average of 2–5 years old reported in other studies [27]. The prevalence of childhood ALL in our cohort was highest among 0–5-year-olds, following similar trends from previously published studies in the literature [26]. Four of our patients had pre-existing genetic conditions such as Down syndrome and cystic fibrosis, which has also been observed in other studies [7]. Based on POG and CCG guidelines [19], our study group was stratified into very high-, high-, standard-, and low-risk groups, resulting in most patients (n = 98) being placed in the standard-risk group.

Out of the total number (*N* = 225) of childhood ALL patients, 61% were treated solely in Kosovo, and 33% were referred abroad due to positive cytogenetics or immediate need for hematopoietic stem cell transplantation. Hematopoietic stem cell transplantation is not legally regulated in Kosovo yet, and therefore is not offered as a service in the Hematology Oncology Department at the Pediatrics Clinic. Childhood ALL patients treated in Kosovo during the study period followed five different treatment protocols, which were available successively. Due to the lack of genetic testing for common polymorphisms and an inability to monitor drug metabolites [28], clinicians modified treatment protocols by reducing the number of administered drugs and doses, aiming to avoid adverse outcomes and reduce mortality rates. Medical research and improvement of treatment protocols are crucial in reducing relapse rates, reducing adverse events, and avoiding secondary complications, contributing to improving OS and quality of life among patients [29]. 

The treatment results of childhood ALL patients in Kosovo are quite promising. After the first course of chemotherapy, more than 61% of the patients acquired full remission, while 11% of them experienced a relapse, in which bone marrow was the most common site (76%), corroborating other studies [30]. The overall mortality of childhood ALL patients was 20%, with 13% due to ALL and 7% due to complications, which is comparable to the findings of Otterman et al. (2019) [31]. The highest mortality rate was observed in the high-risk and very high-risk groups (*p* < 0.001), which corroborates other studies [32].

Globally, the 5-year EFS is considered to be around 70–85%, while the 5-year OS is considered to be 94–95% [33]. In contrast, our cohort revealed lower 5-year EFS and OS rates. Out of all the patients included in our cohort (N = 225), 55% (n = 123) achieved a 2-year EFS, and 40% (n = 90) achieved a 5-year EFS. For OS, 61% of patients (n = 138) achieved a 2-year OS, and 46% (*n* = 104) achieved a 5-year OS. EFS and OS rates in this study are lower compared to studies previously published in developing and developed countries, where OS is approximately 92% [34,35]. We observed highly statistically significant differences in 2-year and 5-year EFS and OS rates when comparing risk groups. The low-risk group exhibited the highest survival rates, whereas patients in the high-risk group had the lowest survival probability. Specifically, the low- and standard-risk groups showed an over 80% probability of achieving 5-year EFS and OS, while the high- and very high-risk groups had 5-year EFS probabilities of 55% and less than 20%, respectively. Their 2-year and 5-year OS rates were around 50 and 60%. 

No statistically significant differences were observed in survival rates when analyzed by age groups or gender, in contrast to the existing literature, which emphasizes that gender is an important prognostic factor and that survival rates are higher among females [36,37]. Also, our study group was homogenous in regards to race and ethnicity, as all participants were white Caucasians and Albanians in origin, while other studies showed significant disparities in the outcome of childhood ALL by race and ethnicity [38]. 

In the treatment protocols of childhood ALL cases treated only in Kosovo, there is a noticeable trend of increasing survival rates over time, correlating with the advancement of protocol modification implemented in the past 15 years. Patients treated under the low-risk protocol and the modified protocol with doxorubicin had the lowest 5-year OS probability (<60%), while patients treated under the standardized AIEOP-BFM-2009 with methotrexate doses lowered to 3 g/m^2^ had a 5-year OS of 90%. Our study showed statistically significant differences in 2-year and 5-year OS rates and in 5-year EFS rates, implying the importance of treatment protocol to overall outcome of childhood ALL patients, as reported previously [39,40]. 

For the period studied, our results are comparable to those of other European medical centers and follow the same trends, which may be due to advanced diagnostics, standardized protocols, increased collaboration with regional and international treatment centers, and good quality of care. Existing studies from various nations and regions have reported better childhood ALL treatment outcomes and prognosis in comparison to our findings [33,34,35], emphasizing there is room for improvement in pediatric healthcare services in Kosovo. These differences can mainly be attributed to disparities in healthcare infrastructure, access to treatment modalities, socio-economic factors affecting patient care and compliance, and differences in treatment protocols and standards of care. Furthermore, MRD measurement has proven to be a robust prognostic factor for response to therapy and is applied in Kosovo during the treatment of ALL patients. However, MRD data were not available to us, and we could not perform association analyses with remission and relapse rates. We realize this can be limitation of our study, and it can be addressed in the future.

In recent years, there has been a significant advancement in the treatment of childhood ALL by considering patients’ genetic profile, deficiency for specific metabolizing genes, response to initial therapy, stratification among different risk groups, and quantification of minimal residual disease, reducing adverse health outcomes and increasing the clinical response [14,28,29,41]. It is essential that these measurements are adopted in Kosovar healthcare to improve childhood ALL treatment outcomes. Finally, ALL clinical outcomes could be improved by lowering toxicity from previous chemotherapeutical regimens and infections through implementing therapeutic novelties in the ALL field, such as intensified chemotherapy schemes, targeted therapies including tyrosine kinase inhibitors and chimeric antigen receptors, and reducing the frequency of blood transfusions [31,41,42,43].

## 5. Conclusions

Despite progress in the field of childhood ALL, efforts among researchers, medical care providers, and supportive teams need to further refine diagnostics and treatment strategies. This would help provide customized care and advance patient clinical outcomes by minimizing long-term side effects while promoting general well-being.

## Figures and Tables

**Table 1:** Demographic characteristics of the study population. Data comprise information from pediatric patients with acute lymphoblastic leukemia (ALL, N = 225) diagnosed with ALL between 2008 and 2023 in the Department of Pediatric Hematology-Oncology, University Clinical Center of Kosovo.

Time Period 2008–2023	Number of Patients (%)
Childhood ALL patients	225 (100)
Gender	
Female	91 (40)
Male	134 (60)
Age at diagnosis	
0–5 years old	98 (43)
5–10 years old	64 (28)
>10 years old	63 (28)
Comorbidities	
Down syndrome	3 (1.3)
Cystic fibrosis	1 (0.4)
Risk group	
Very high-risk group	35 (16)
High-risk group	81 (36)
Standard risk group	98 (43)
Low-risk group	11 (5)

ALL—Acute lymphoblastic leukemia.

**Table 2:** Characteristics of risk groups classifying pediatric patients with acute lymphoblastic leukemia (ALL). Data comprise information from pediatric patients with ALL (N = 225) diagnosed between 2008 and 2023 at the Department of Pediatric Hematology-Oncology, University Clinical Center of Kosovo. Statistical analysis was performed by chi-squared test. Statistical significance was evaluated at an alpha level (α) of 0.05.

	Risk Group				
Characteristics	Very High *n*	High *n*	Standard *n*	Low *n*	*p* Value
Age group					
<5 years	13	24	56	5	*p* < 0.001
5–10 years	6	16	37	5	
>10 years	16	41	5	1	
Gender					
Female (N = 91)	12	29	47	3	*p* = 0.2246
Male (N = 134)	23	52	51	8	
Comorbidities					
Yes	0	2	1	1	*p* = 0.6848
No	35	79	97	10	

**Table 3:** Treatment protocols used to treat pediatric patients with acute lymphoblastic leukemia (ALL) and/or country of treatment (N = 225).

Treatment Center	Number of Patients (%)
Kosovo	138 (61)
Kosovo + Abroad	13 (6)
Treatment protocols	
Low-risk Kosovo protocol for childhood ALL	43 (28)
Modified protocol with doxorubicin	16 (11)
Modified protocol with doxorubicin + delayed intensification	12 (8)
Modified AIEOP-BFM - 2009 protocol with MTX 3 g/m^2^	58 (38)
Modified AIEOP-BFM - 2009 protocol with MTX 3.5 g/m^2^	22 (15)
Only abroad	74 (33)
Turkey	40 (54)
Italy	14 (19)
Austria	8 (11)
Belgrade	3 (4)
Germany, North Macedonia, Geneva	6 (8)
France, Croatia, Sweden	3 (4)

**Table 4:** Remission, relapse, and mortality rates of pediatric patients with acute lymphoblastic leukemia (ALL).

Patients	N = 225
Monitored	n = 203
Lost to follow-up	n = 22
Treatment course (n total = 203)	n (%)
Remission (after first cycle of chemotherapy)	123 (61)
Hematopoietic stem cell transplantation	19 (9)
In first remission	18 (9)
In second remission	18 (9)
Relapse	23 (11)
Bone marrow	17 (76)
Testes	2 (8)
Retina	2 (8)
CNS infiltration	2 (8)
Mortality rate	
Mortality rate for cases treated in Kosovo (N tot = 141)	28 (20)
Mortality rate for cases referred abroad (N tot = 60)	15 (25)
Cause of death	
Death due to primary diagnosis	18 (13)
Death due to secondary complications	10 (7)
Sepsis	6
Acute respiratory distress syndrome	2
Thrombophlebitis	2
Meningitis	1
Intracranial bleeding	1
Pseudomonas aeruginosa infection	1
Liver failure	1
Paralytic ileus	1
Parotitis	1

**Table 5:** Two-year and five-year overall (OS) and event-free survival (EFS) analyses based on age group, risk group, gender, and treatment protocol. Data comprise information from pediatric patients with acute lymphoblastic leukemia (ALL, *N* = 225), diagnosed with ALL between 2008 and 2023 at the Department of Pediatric Hematology-Oncology, University Clinical Center of Kosovo. Statistical analysis was performed using a log-rank test. Statistical significance was evaluated at an alpha level (α) of 0.05.

	2yOS ± SD	5yOS ± SD	*p*-Value	2yEFS ± SD	5yEFS ± SD	*p*-Value
Age at diagnosis						
0–5	18.66 ± 0.95	44.97 ± 2.53	2y OS *p* = 0.698 5y OS *p* = 0.693	18.01 ± 0.95	41.68 ± 2.63	2y EFS *p* = 0.573 5y EFS *p* = 0.353
5–10	18.39 ± 1.20	44.52 ± 3.30		17.48 ± 1.24	39.16 ± 3.36	
>10	17.86 ± 1.22	41.90 ± 3.27		16.82 ± 1.20	35.81 ± 3.36	
Risk group						
Very high risk	15.25 ± 1.79	34.36 ± 4.87	*p* < 0.001	10.25 ± 1.47	11.36 ± 1.83	*p* < 0.001
High risk	17.04 ± 1.10	39.71 ± 2.97		16.66 ± 1.08	36.88 ± 2.96	
Standard risk	20.35 ± 0.83	50.41 ± 2.19		20.35 ± 0.83	50.41 ± 2.19	
Lower risk	20.47 ± 2.25	49.56 ± 6.65		20.47 ± 2.25	49.56 ± 6.65	
Gender						
Female	19.31 ± 0.92	46.37 ± 2.53	2y OS *p* = 0.188 5y OS *p* = 0.181	18.63 ± 0.92	42.70 ± 2.67	2y EFS *p* = 0.134 5y EFS *p* = 0.116
Male	17.71 ± 0.85	42.34 ± 2.30		16.77 ± 0.86	37.06 ± 2.33	
Treatment protocol						
Low risk	16.53 ± 1.51	36.44 ± 4.02	*p* < 0.001	16.09 ± 1.46	33.27 ± 3.96	2y EFS *p* = 0.30 5y EFS *p* = 0.010
Intermediate risk protocol with doxorubicin	14.56 ± 2.69	34.81 ± 7.14		14.56 ± 2.69	34.81 ± 7.14	
Doxorubicin+ delayed intensification	18.5 ± 2.75	45.50 ± 7.25		18.16 ± 2.72	42.16 ± 7.39	
AIEOP-BFM-2009 3 g MTX	21.46 ± 0.90	53.12 ± 2.44		20.01 ± 1.02	46.72 ± 2.98	
AIEOP-BFM-2009 3.5 g MTX	22.04 ± 0.93	NA *		22.04 ± 0.93	NA *	

* The AIEOP-BFM-2009 3.5 g MTX treatment protocol started to be implemented in 2022.

## Data Availability

The data presented in this study are available on request from the corresponding author due to privacy and ethical restrictions.

## Supplementary Materials

The following supporting information can be downloaded at: https://www.mdpi.com/article/10.3390/cancers16111988/s1, Detailed childhood ALL treatment protocols followed in Kosovo from 2008 to 2023; Figure S1. The 2-year and 5-year event-free survival (EFS) by age group, risk group, and treatment protocol; Figure S2. The 2-year and 5-year overall survival (OS) by age group, risk group, and treatment protocol; Table S1. Classification of the patients into risk groups according to their treatment protocols and institutions; Table S2. Mortality rates and risk groups of childhood ALL cases treated in Kosovo and abroad. Statistical analysis was performed using the chi-squared test. The threshold for significance was *p* < 0.05; Table S3. Mortality rates and treatment protocols for childhood ALL patients treated solely in Kosovo. Statistical analysis was performed using the chi-squared test. The threshold for significance was *p* < 0.05.
