# Supplementary material for: Prevalence and Treatment Outcomes of Childhood Acute Lymphoblastic Leukemia in Kosovo
Source: Cancers (Basel). 2024 May 23;16(11):1988. doi: 10.3390/cancers16111988 (PMC11171310; doi:10.3390/cancers16111988)
Supplement: Supplementary file 1 [file cancers-16-01988-s001.zip › cancers-3007973-supplementary.pdf]

Supplementary Materials: Prevalence and Treatment Outcomes of Childhood Acute Lymphoblastic Leukemia in Kosovo

Flaka Pasha [1,2], Dunja Urbančič [1], Rufadie Maxhuni [3], Shaip Krasniqi [2], Violeta Grajçevci Uka [3,*], Irena Mlinarič-Raščan [1,*]

[1] University of Ljubljana, Faculty of Pharmacy, Askerceva 7, 1000 Ljubljana, Slovenia
[2] Department of Pharmacology with Toxicology, and Clinical Pharmacology, Faculty of Medicine, University of Prishtina "Hasan Prishtina," 10000, Kosovo
[3] Department of Hematology-Oncology, Pediatric Clinic, University Clinical Center of Kosovo, Prishtine, 10000, Kosovo
* Correspondence: Email: irena.mlinaric@ffa.uni-lj.si, Phone number: +386 1 4769 500 (I.M.-R.); Email: violetagrajqevci@hotmail.com, Phone number: +383 44 263 741 (V.G.U.)

SI- Detailed childhood ALL treatment protocols followed in Kosovo from 2008 to 2023

During the investigated time period, five different treatment protocols were used to treat children with ALL. Treatment protocols are presented in detail below:

a. The Low-Risk Kosovo Protocol for ALL was followed from 2008 to June 2012. In the Low-Risk Kosovo Protocol for ALL patients, during the Induction Phase (Day 1–42) patients started with 60mg/m^2/day of oral Prednisone, divided into intervals of eight hours. Prednisone was slightly increased to a steady dose on the first week and reduced gradually during the last week of treatment. Further, patients received bolus Vincristine of 1.5mg/m^2 in day 7, 14, 21, 28, 35, and 42. Intramuscular L- asparaginase of 6000 IU/m^2 was administered on day 23 and 30 of treatment. While, intrathecal Methotrexate adjusted for age was given on days 11 and 39. The induction phase was concluded with a bone marrow puncture on day 42. After having completed the induction phase protocol, the children did not receive any treatment for 1–2 weeks, so their blood cells could recover. The Low-Risk Kosovo protocol for childhood ALL patients, was followed by the consolidation phase, consisting of 35 days in total. Patients would take daily oral 6- Mercaptopurine of 50mg/m^2/day, followed with intramuscular L- asparaginase of 6000 UI/m^2, and intrathecal Methotrexate age-adjusted dose on days 8, 15, and 22. In the maintenance phase, patients would take oral Prednisone of 60mg/m^2/day on the first day of the week, divided into intervals of eight hours, Vincristine of 1.5mg/m^2, and intrathecal Methotrexate adjusted for age. In the following days of the week, patients took oral 6- Mercaptopurine of 50mg/m^2/day and Methotrexate of 20mg/m^2.

b. The Modified Protocol with Doxorubicin was followed from July 2012 to December 2013. The modified protocol with Doxorubicin, resembled the Low-Risk Kosovo protocol for childhood ALL with minor changes, where instead of Prednisone patients received oral Dexamethasone of 10mg/m^2/day, and intravenous Doxorubicin of 25 mg/m^2 was added on days 8 and 15th of the treatment.

c. The Modified Protocol with Doxorubicin and Delayed Intensification was followed from 2014–2015. During the induction phase, patients received oral Dexamethasone of 10mg/m^2/day on days 1–7, and 15–21, and then followed with intravenous Vincristin of 1.5mg/m^2, Doxorubicin 25mg/m^2, and L-Asparaginase of 6000 UI/m^2 on days 1, 8, and 15. The protocol further included the delayed intensification, where intravenous Cyclophosphamides of 1000mg/m^2 on day 29 was applied, followed by intravenous or subcutaneous Cytarabine of 75mg/m^2 on days 31–34, and 38–41. Oral 6-Mercaptopurine of 50mg/m^2/day was given from the 29th–34th day, and an intrathecal age-adjusted dose of Methotrexate was applied on days 1, 31, and 38. A bone marrow aspiration was performed at the end of the protocol to measure the residual disease.

d. A Modified AIEOP-BFM ALL 2009 Protocol with 3g/m^2 of Methotrexate and 5000 UI/m^2 of L- Asparaginase was followed from 2016 to 2021.

e. A Modified AIEOP-BFM ALL 2009 Protocol with 3.5g/m^2 of Methotrexate and 5000 UI/m^2 of L-Asparaginase was followed from 2022 and is ongoing.

Table S1. Classification of the patients into risk groups according to their treatment protocols and institutions.

Characteristics	Risk group			
	Very high<br>*n*	High<br>*n*	Standard<br>*n*	Low<br>*n*
**Treatment protocol**				
Low-Risk Kosovo Protocol	8	13	20	2
Modified Protocol with Doxorubicin	0	7	9	0
Modified Protocol with Doxorubicin and delayed intensification	1	3	8	0
AIEOP BFM with 3g/m^2 MTX	6	19	25	8
AIEOP BFM with 3.5g/m^2 MTX	0	6	15	1
Abroad	20	33	21	0
**Treatment Institution**				
Kosovo	10	42	75	11
Kosovo + abroad	5	6	2	0
Abroad	20	33	21	0

MTX – methotrexate.

Table S2. Mortality rates and risk group of childhood ALL cases treated in Kosovo and abroad. Statistical analysis was performed by chi-squared test. The threshold for significance was $p<0.05$.

	Risk group				
**Characteristics**	**Very high**	**High**	**Standard**	**Low**	
**Mortality rate**	*n*	*n*	*n*	*n*	
Kosovo and Kosovo+ abroad	7	16	4	1	***P* <0.001**
Abroad	8	6	0	1	

Statistical analysis revealed a significant correlation between mortality rates and risk group stratification (p<0.001) in Kosovo. The highest mortality rate was observed in high-risk and very high-risk groups. Bonferroni post hoc test correction, showed significant differences in standard, high-risk, and very high-risk groups in comparison to mortality rates.

Table S3. Mortality rates and Treatment protocols for childhood ALL patients treated solely in Kosovo. Statistical analysis was performed by chi-squared test. The threshold for significance was $p<0.05$.

	Treatment protocols					
**Characteristics**						
**Mortality rate**	**Low risk**<br>*n*	**Modified Doxorubicin**<br>*n*	**Modified Doxorubicin + Delayed intensification**<br>*n*	**AIEOP BFM ALL 2009 3g/m^2 MTX**<br>*n*	**AIEOP BFM ALL 2009 3.5g/m^2 MTX**<br>*n*	***P* <0.001**
Kosovo	18	4	1	4	1	

With regard to the treatment protocols followed in Kosovo, the highest mortality rate was observed in patients under Low-Risk Kosovo Protocol (n=18), followed by those treated with modified AIEOP- BFM with 3g/m^2 of Methotrexate (n=4), and Modified Protocol with Doxorubicin (n=4). The lowest mortality rate was observed among patients treated with Modified Protocol with Doxorubicin with Delayed Intensification, and AIEOP- BFM with 3.5g/m^2 of Methotrexate. Bonferroni post hoc test correction, showed significant differences only in Low-Risk Kosovo treatment protocol and the mortality rate.

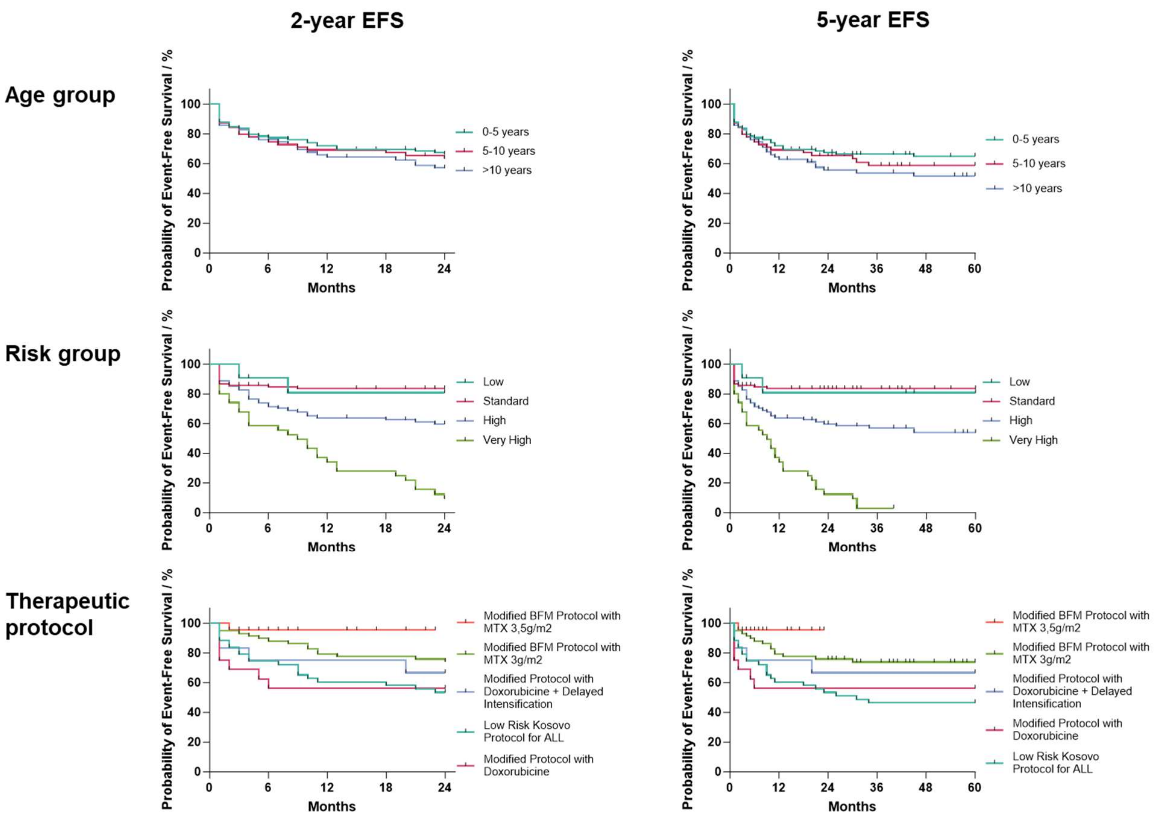

Figure S1. The 2-year and 5-year event-free survival (EFS) by age group, risk group, and treatment protocol.

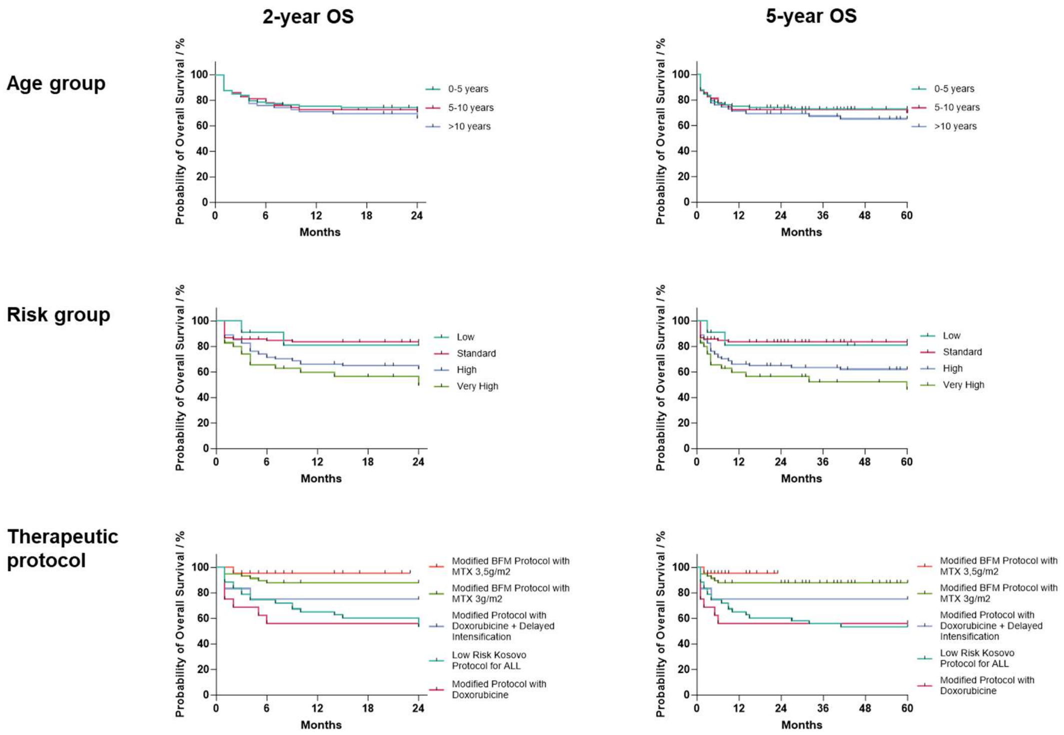

Figure S2. The 2-year and 5-year overall survival (OS) by age group, risk group, and treatment protocol.
